# Engineering T-cells with chimeric antigen receptors to combat hematological cancers: an update on clinical trials

**DOI:** 10.1007/s00262-022-03163-y

**Published:** 2022-02-23

**Authors:** Maria Ormhøj, Hinrich Abken, Sine R. Hadrup

**Affiliations:** 1 Department of Health Technology, Technical University of Denmark, Lyngby, Denmark; 2 Department Genetic Immunotherapy, Leibniz Institute for Immunotherapy, Universitätsklinikum Regensburg, Regensburg, Germany

**Keywords:** Chimeric antigen receptor, Hematological cancer, Clinical trial

## Abstract

Chimeric antigen receptor (CAR) redirected T-cells has shown efficacy in the treatment of B-cell leukemia/lymphoma, however, high numbers of relapses occur due to loss of targeted antigen or intrinsic failure of the CAR T-cells. In this situation modifications of the basic strategy are envisaged to reduce the risk of relapse, some of them are in early clinical exploration. These include simultaneous targeting of multiple antigens or combination of CAR T-cell therapy with other treatment modalities such as checkpoint inhibitors. The review evaluates and discusses these modified advanced therapies and pre-clinical approaches with respect to their potential to control leukemia and lymphoma in the long-term.

## The molecular structure and function of CARs

**Fig. 1:**
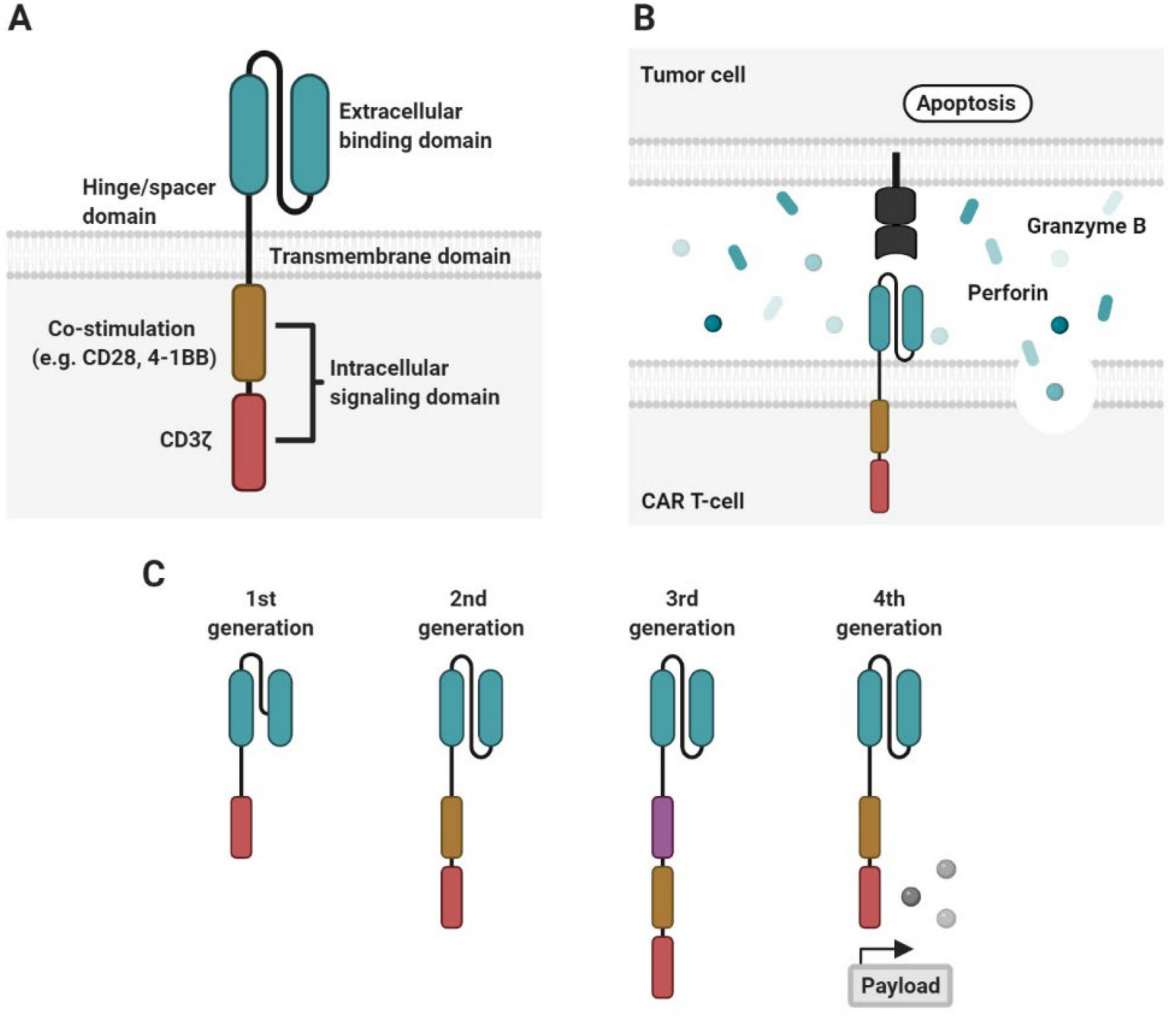
CAR structure and function: **A** T-cells can be endowed with a new specificity against a surface tumor antigen through genetic introduction of a chimeric antigen receptor (CAR). CARs are fusion proteins composed of (i) extracellular binding domain, often consisting of a single chain variable fragment (scFv) derived from a monoclonal antibody, (ii) a hinge/spacer domain, (iii) a transmembrane domain and (iv) an intracellular signaling domain. **B** Recognition of cognate antigen by the CAR T-cell leads to cytolytic degranulation with release of perforin and granzyme *B*, a major mechanism for CAR T-cell mediated killing. **C** Traditionally, CARs are divided into three generations based on the number of incorporated co-stimulatory domains. Lately, a fourth generation of CAR T-cells has emerged called T-cells redirected for universal cytokine killing (TRUCKs), which combine the expression of a CAR with inducible secretion of a transgenic payload

T-cells genetically engineered to express a chimeric antigen receptor (CAR) directed against the pan B-cell marker CD19 has shown tremendous potential against hematological malignancies leading to the approval of several CD19 CAR T-cell therapies. CARs are fusion proteins composed of four domains, (i) an extracellular binding domain, often consisting of a single chain fragment of variable region (scFv) derived from a monoclonal antibody, (ii) a hinge/spacer domain, (iii) a transmembrane domain and (iv) an intracellular signaling domain (Fig. 1A) [1]. Once CAR T-cells engage cognate antigen on the target cell, the CAR molecules cluster on the cell surface forming an immunological synapse (IS), leading to cytolytic degranulation with release of perforin and granzyme B which induces apoptosis of the targeted cancer cell (Fig. 1B). It has been demonstrated that both CD4+ and CD8+ CAR T-cells are capable of direct lysis of tumor cells, albeit with different kinetics attributed to their differences in granzyme B content [2].

Traditionally, CARs are divided into three generations based on the number of incorporated co-stimulatory domains. Where 1st generation CARs only harbor a primary activation domain like the CD3ζ or FcεRIγ signaling chain domain, 2nd and 3rd generation CARs also include one or two co-stimulatory domains, respectively, besides the primary activation domain (Fig. 1C). The CD3ζ activation domain alone is not sufficient to drive optimal T-cell proliferation and cytokine production in patients. However, with the incorporation of co-stimulatory domains efficacy and persistence of 2nd and 3rd generation CAR T-cells have improved dramatically. Typically, 4-1BB or CD28 has been used as co-stimulatory domains in CAR T-cells, but other members of the costimulatory signaling families are also used. In general, signaling through CD28 leads to rapid T-cell activation with highly elevated cytokine secretion, aerobic glycolysis and reduced T-cell persistence [3–5]. In contrast, signaling through 4-1BB leads to a slower T-cell response and lower cytokine secretion, but promotes oxidative metabolism and prolonged persistence [4, 5]. Lastly, T-cells redirected for universal cytokine killing (TRUCKs) combine the expression of a CAR with CAR activation induced secretion of a transgenic payload like cytokines; these engineered T-cells have been named the 4th generation of CARs [6]. The TRUCK approach has further been built upon to include secretion of enzymes that modulate the tumor matrix, secretion of checkpoint inhibitors, secretion of bispecific T-cell engagers (BiTEs) or expression of co-stimulatory ligands, all with the goal of augmenting CAR T-cell efficacy and/or to modulate the immune environment in the targeted tumor tissue.

## Emerging CAR-based strategies for targeting hematological malignancies

### Acute lymphoblastic leukemia

CD19 is considered the cardinal marker for B-cells and is involved in establishing intrinsic B-cell signaling thresholds by modulating both B-cell receptor (BCR) dependent and independent signaling. The vast majority of clinical studies investigating CAR T-cell therapy in B-cell malignancies have been focused on targeting CD19 (Table 1). These studies have reported high response rates and pioneered the approval of the first anti-CD19 CAR T-cell therapy, tisagenlecleucel, a second generation 4-1BB based CAR for relapsed/refractory (r/r) ALL in children and young adults [7]. Despite the encouraging results obtained for anti-CD19 CAR T-cells roughly 1/3 of patients relapse with CD19-negative disease. Consequently, development of new CAR T-cell therapies targeting alternative antigens that ideally are closely associated with B-cell development and/or malignant transformation are warranted. One such emerging antigen is CD22 which, like CD19, is broadly expressed during B-cell development. The potential of the strategy is shown in several clinical studies with CAR T-cells directed against CD22 that induced remissions in both CD19–CAR treated naïve patients and patients with previous relapse with CD19-negative tumors after CD19 CAR T-cell therapy [8, 9]. To reduce the risk of relapse current clinical investigations are looking into the efficacy of combining CAR T-cell products targeting both CD19 and CD22 (e.g., NCT03185494, NCT03289455).

**Table 1:** CAR T-cell strategies for hematological cancer

Target	CAR format	Co-stim domain	Nr. of evaluated patients	CR (%)	CRS grade ≥ 3 (%)	Neurotox. grade ≥ 3 (%)	Clinical trial identifier	References
*Acute lymphoblastic leukemia*								
CD19^#^	Single	4-1BB	63	63	49	18	NCT02435849	[7]
CD22	Single	4-1BB	21	57	0	N/A	NCT02315612	[8]
CD19/CD22	Tandem	4-1BB	6	100	0	0	NCT03185494	[10]
CD19/CD22	Dual	OX40/4-1BB	7*	100	0	0	NCT03289455	[11]
CD19/CD123	Dual	4-1BB/4-1BB	–	–	–	–	–	[12]
CD19/CD123	Pooled	CD28 + CD27	3	100	0	0	NCT03125577	[13, 14]
CSPG4	Single	CD28	–	–	–	–	–	[15]
BAFF-R	Single	4-1BB	–	–	–	–	NCT04690595	[16, 17]
*Chronic lymphocytic leukemia*								
CD19	Single	4-1BB	3	66	–	–	NCT01029366	[18, 19]
CD19 + ibrutinib	Single	4-1BB	19	83	0	26	NCT01865617	[20]
CD19/CD20	Tandem	4-1BB	22***	64	5	14	NCT03019055	[21]
κ light chain	Single	CD28	16****	12.5	0	–	NCT00881920	[22]
FcμR	Single	CD28	–	–	–	–	–	[23]
*Multiple myeloma*								
BCMA^#^	Single	4-1BB	128	32.8	5	3	NCT03361748	[24]
BCMA*****	Tandem	4-1BB	17	76.4	53.8	–	NCT03090659	[25]
BCMA/TACI	Single*****	CD28 + OX40	11	0	0	0	NCT03287804	[26, 27]
CD19 + ASCT	Single	4-1BB	11	1	0	0	NCT02135406	[28, 29]
CD38	Single	4-1BB	–	–	–	–	–	[30, 31]
CD138	Single	4-1BB	5	0	–	–	NCT01886976	[32]
CS1******	Single	CD28	–	–	–	–	NCT03958656	[33]
GPRC5D	Single	4-1BB	–	–	–	–	NCT04555551	[34]
Integrin β7	Single	CD28	–	–	–	–	–	[35]
NKG2D ligands	Single*******	None	12	0	0	–	NCT02203825	[36]
CS1/BCMA	Tandem	4-1BB	–	–	–	–	–	[37]
CD19/BCMA	Pooled	4-1BB/4-1BB	21	57	5	–	ChiCTR-OIC-17011272	[38]
*B-cell lymphoma*								
CD19^#^	Single	CD28	68	59	15	31	NCT02601313	[39]
CD30	Single	4-1BB	18	0	0	0	NCT022595	[40]
CD37	Single	4-1BB	–	–	–	–	NCT04136275	[41]
CD79b	Single	CD28-4-1BB	–	–	–	–	ChiCTR-OPN-16008526	[42]
CD79b/CD19	Tandem	4-1BB	–	–	–	–	–	[43]
*T-cell lymphoma*								
TRBC1	Single	CD28 + OX40	–	–	–	–	NCT03590574	[44]
CD30	Single	CD28	1	100	0	0	NCT02690545	[45]
CD37	Single	4-1BB	–	–	–	–	NCT04136275	[41]

^#^Approved product; –No data available; N/A not available; *10 patients treated but only 7 evaluable at cut-off-date; **3 patients with CLL, remaining patients diagnosed with other r/r B-cell malignancies; ***2 patients with CLL, remaining patients diagnosed with other r/r B-cell malignancies; ****Biepitopic; *****Utilizes full length APRIL domain as binder; ******Suicide switch; *******Utilizes full length extracellular NKG2D domain as binder. *CSPG4* Chondroitin Sulfate Proteoglycan-4, *BAFF-R* B-cell activating factor receptor, *BCMA* B-cell maturation antigen; *TACI* Transmembrane activator and CAML interactor; *GPRC5D* G protein-coupled receptor class C group 5 member-D*TRBC1* T cell receptor beta constant-1

Other combinatorial strategies include co-targeting of CD19 and CD123 since CD123 is expressed on CD19-negative clones, most likely present before initiation of CD19 CAR T-cell therapy and on leukemic-initiating cells [12]. In a recent report, CAR T-cells directed against chondroitin sulfate proteoglycan-4 (CSPG4) showed in vitro efficacy against positive target cells [15]. CSPG4 is expressed on the surface of mixed-lineage leukemia-1 (MLL1)-rearranged leukemic cells, which are associated with a poorer prognosis compared to non-MLL1 rearranged ALL [15]. Other targets under investigation include B-cell activating factor receptor (BAFF-R) [16], a B-cell specific antigen critical for normal B-cell survival. Given the critical role in both healthy and malignant B-cell survival, targeting of BAFF-R is envisioned to reduce the number of patients relapsing with BAFF-R-negative escape variants after therapy. Clinical testing of BAFF-R CAR T-cells are underway (NCT04690595).

### Chronic lymphocytic leukemia

Despite the impressive results of CD19 CAR T-cells in ALL, similar efficacy has not been observed in chronic lymphocytic leukemia (CLL). The reported lower efficacy of CD19 CAR T-cells in CLL may be attributed to an initially reduced functional state of the patient T-cells used for manufacturing. T-cells from CLL patients have previously been shown to have impaired functional capacity and display an exhausted phenotype. Based on this, several groups are focused on augmenting CAR T-cell efficacy in CLL (Table 1). One such strategy is based on combining CD19 CAR T-cell therapy with an irreversible inhibitor of Bruton’s tyrosine kinase (BTK), known as Ibrutinib [46]. BTK is involved in the pathogenesis of CLL since constitutive activation of the kinase is linked to constitutive activation of the BCR signaling pathway and consequently increased survival and proliferation of malignant cells. Besides directly affecting CLL cells, Ibrutinib also exerts immunomodulatory effects on T-cells through inhibition of BTK and IL-2-inducible T-cell kinase (ITK). Inhibition of these kinases increases T-cell numbers and enhances persistence of activated T-cells in patients. Combining CD19 CAR T-cell therapy with Ibrutinib compared to Ibrutinib or CD19 CAR T-cells alone significantly increased the overall survival of mice in a xenograft model of CLL [46]. Additionally, ongoing clinical trials evaluating the effect of the Ibrutinib/CD19 CAR T-cell combination in CLL have so far been promising with increased response rates compared to previous studies with CD19 CAR T-cells alone [20] (NCT01865617). An alternative approach to circumvent the diminished functionality of CAR T-cells from CLL patients could be utilizing allogeneic healthy donor T-cells as a source for CAR T-cell manufacturing.

Besides CD19, other targets such as CD20, CD22 and various combinations thereof are currently being explored as CAR targets in CLL. The efficacy of CAR T-cells directed against *κ* light chain has been tested in patients with CLL with remissions in a fraction of patients [22] (NCT00881920). Due to the clonal nature of cancer cells in CLL, authors took advantage of the fact that malignant cells only express either the *κ* or the *λ* light chain. In this way, healthy B-cells expressing the *λ* light chain could be spared, while *κ* light chain-expressing malignant and healthy cells were killed. A similar approach, with the purpose of sparing healthy B-cells from CAR T-cell mediated killing, was attempted by targeting the IgM Fcμ receptor (FcμR) [23]. The FcμR is highly expressed on malignant CLL cells compared to healthy B-cells. In this pre-clinical study, T-cells derived from CLL patients were able to eradicate their autologous malignant CLL tumors without cytotoxicity against their healthy B-cell counterparts.

### Multiple myeloma

In regard to multiple myeloma (MM), B-cell maturation antigen (BCMA) remains the most explored target for CAR T-cell therapy. In recent years, several clinical trials have been published and provided promising outcomes with overall response rates (ORR) of more than 80% [47, 48]. Recently, the first anti-BCMA CAR T-cell therapy (Idecabtagene vicleucel) was approved by the FDA. Despite these encouraging results, relapse with BCMA-negative tumors have been reported in several studies clearly highlighting antigen-escape as an emerging obstacle for the curative potential of anti-BCMA CAR T-cells in myeloma.

Next generation BCMA CAR T-cells are currently in clinical evaluation to address some of the above limitations (Table 1). Shah et al*.* [49] reported initial results from a phase 1 study testing bb21217, an anti-BCMA-4-1BB-CD3ζ CAR T-cell product (NCT03274219). Unlike previous studies with similar products from Bluebird Bio, the bb21217 product is manufactured in the presence of a PI3K inhibitor during ex vivo culture with the aim of enriching the final product for T-cells with a memory phenotype. Several pre-clinical studies suggested increased persistence and efficacy of such a CAR T-cell product. In total, 83% of patients treated on this protocol had a clinical response with emerging data demonstrating long-term persistence of CAR T-cells in long-term responders. However, longer follow-up time and inclusion of additional patients are needed to support initial findings and establish improved persistence with this product.

Poseida Therapeutics recently reported initial results from their ongoing phase 1/2 study (NCT03288493) [50]. Instead of utilizing a scFv as binder, their P-BCMA-101 construct is based on an anti-BCMA centyrin. Centyrins are small non-antibody proteins, derived from human consensus tenascin FN3 domains, which can be engineered to bind specific targets with high affinity and specificity [51]. The P-BCMA-101 CAR T-cell manufacturing utilizes the piggyBac (PB) transposon system instead of viral gene transfer which is claimed to enrich for a population of CAR T-cells with a memory phenotype. Based on an ongoing phase 1/2 trial, P-BCMA-101 has shown an ORR of 57% (NCT03288493). Recently, development of CAR+ T-cell lymphoma in two patients receiving PB engineered anti-CD19 CAR T-cells was reported [52, 53]. The underlying mechanism for malignant T-cell transformation was not apparently caused by insertional mutagenesis due to the CAR gene but rather as a consequence of the electroporation procedure.

Due to the reported BCMA-negative relapses, other targets including Igκ light chain, CD19 and CD138 are currently being pursued as targets in clinical trials [54]. Recently, CD38 has also emerged as alternative target for CAR T-cells in MM. Although highly expressed on malignant myeloma cells, CD38 is also expressed albeit at lower levels on natural killer (NK) cells, monocytes and some T-cells during infection. Therefore, targeting CD38 poses a risk for on-target/off-tumor toxicity and could hamper an ongoing immune response during infection. Utilizing a high-affinity scFv as binder, CD38 CAR T-cells led to killing of both high and dim CD38-expressing target cells. Drent et al. [30] showed that reducing the affinity of the scFv could spare target cells with a dim CD38 expression but maintain cytolytic activity against MM tumor cells with high CD38 expression. Because of the high risk of on-target/off-tumor toxicity occurring with targeting CD38, Drent and colleagues introduced a “safety-switch” in their affinity-optimized CD38 CAR T-cell product [31]. The safety switch allows only for CD38 CAR expression in T-cells in the presence of doxycycline due to an engineered Tet-on system; in absence of doxcycycline no CAR is expressed. Trials exploring anti-CD38 CAR T-cells as therapy for MM are currently in the recruitment phase (e.g., NCT03464916). Additional proposed targets for CAR T-cell treatment of MM include CS1 [55], integrin β7 [35], and *G* protein-coupled receptor class *C* group 5 member-D (GPRC5D) [34].

CS1 is a surface glycoprotein that is highly and uniformly expressed on MM cells, but also on other immune cells. A phase 1 study recruiting at City of Hope Medical Center is aiming at evaluating the safety and activity of CS1-directed CAR T-cells (NCT03710421). Another approach to target multiple myeloma is to target the MM stem cell compartment. The stem cells are classically defined as CD19 + CD38low/ − which makes CD19 an attractive target for preventing disease recurrence after elimination of the differentiated plasma cells. Results from a clinical trial targeting myeloma stem cells with CD19 CAR T-cell therapy were published from Perelman School of Medicine, University of Pennsylvania [28, 29] (NCT02135406). In total, 11 patients were treated with anti-CD19 CAR T-cell therapy and high-dose melphalan followed by autologous stem cell transplantation. One patient experienced a complete response (CR) with additional 8 patients experiencing a meaningful clinical response classified as very good partial response (VGPR) or partial response (PR). A recent strategy combining CD19 and BCMA targeted CAR T-cell therapy has been published [38]. In this trial conducted at Affiliated Hospital of Xuzhou Medical University, China, 57% of patients experienced a CR with a median follow up time of 179 days (ChiCTR-OIC-17011272). Further studies are warranted to fully understand the mechanisms reported in these trials.

To identify a target selective for MM, Hosen et al. [35] screened more than 10,000 monoclonal antibodies raised against MM cells and identified one clone specifically recognizing an epitope selectively exposed and accessible in the active conformation of integrin β7. Expression of this conformation of integrin β7 was validated in a large cohort of MM patients. Repurposing this clone into a scFv format and CAR binder, anti-β7 CAR T-cells showed specific anti-myeloma efficacy without damaging normal tissue expressing the inactive conformation of integrin β7. Interestingly, authors also showed the ability of these CAR T-cells to recognize CD19+ putative myeloma stem cells [56].

GPRC5D was recently identified as potential target in MM with similar distribution as BCMA [34]. Authors screened a total of 42 different CAR constructs differing in anti-GPRC5D scFv and spacer length with respect to in vitro anti-tumor efficacy and low tonic signaling. In vivo testing of the most effective construct showed complete tumor clearance in both bone marrow tropic MM and BCMA-negative escape MM xenograft models. An interesting approach to limit antigen-escape in MM is utilizing the natural ligand A proliferation-inducing ligand (APRIL) as CAR binding moiety [26], because APRIL is able to bind BCMA but also transmembrane activator and CAML interactor (TACI), both of which are highly expressed on MM cells. A clinical study investigating the safety and efficacy of APRIL-based CAR T-cells is ongoing (NCT03287804). Recently it was shown that converting the APRIL binder from a monomeric into a trimeric form enhances the in vivo efficacy against both BCMA-positive and negative MM, the latter through binding of TACI, in pre-clinical xenograft models [57].

Efforts to augment BCMA CAR T-cell therapy are underway. BCMA is cleaved from tumor cells producing high concentrations of soluble BCMA, thus posing a risk for BCMA-negative relapse and blocking of anti-BCMA CAR T-cell target binding. To circumvent such issues, recent research has focused on combining BCMA CAR T-cells with *γ*-secretase inhibitors to stop BCMA shedding [58]. The strategy is currently being tested in a phase 1 clinical trial (NCT03502577). It has been shown that ex vivo culture of anti-myeloma CAR T-cells in the presence of lenalidomide improves CAR T-cell effector functions [59]. Lenalidomide is a small immune modulatory drug with both direct anti-tumor activity and T-cell modulatory properties. Upon T-cell receptor (TCR) activation, lenalidomide increases phosphorylation of the cytoplasmic tail of CD28, thereby enhancing downstream signaling leading to enhanced functional and proliferative capacity of T-cells. The strategy is currently being pursued in a clinical trial sponsored by Memorial Sloan Kettering Cancer Center (MSKCC) in collaboration with Juno Therapeutics (NCT03070327).

### B-cell lymphoma

Like for CLL, the response rates observed in ALL after CD19 CAR T-cell therapy are not recapitulated in lymphoma patients. Despite this, clinical benefit of targeting CD19 has been established leading to current approval of four CD19 targeting CAR T-cell therapies; tisagenlecleucel, axicabtagene ciloleucel, Lisocabtagene maraleucel and recently brexucabtagene autoleucel for different subtypes of B-cell lymphoma (Table 1).

CD20 and CD22 are also being explored as targets for CAR T-cell therapy in lymphoma. CAR T-cells targeting CD30 are currently in clinical investigation for the treatment of Hodgkin Lymphoma (HL) (NCT03049449) [60]. Several concerns were initially raised when targeting CD30, as this molecule is also expressed on hematopoietic stem cells (HSCs), as well as activated T and B-cells. Lysis of HSCs could cause impaired hematopoiesis. Importantly, targeting CD30 by CAR T-cells led to tumor cell lysis, but did not induce killing of HSCs [61]. The authors attributed these findings to an HSC CD30 expression level below the required threshold for CAR T-cell activation and intrinsic mechanisms in the HSCs, such as high levels of the granzyme B inhibitor PI-9 that protected cells from lysis. This is consistent with published results from clinical trials showing anti-CD30 CAR T-cells to be safe and with anti-tumor efficacy [40, 60]. Similar anti-tumor efficacy of CD19 and CD79b (a pan B-cell marker) CAR T-cells in models of mantle cell lymphoma (MCL) was reported [62]. Sustained response in a pre-clinical MCL xenograft model after treatment with CD79b CAR T-cells in a single or dual format combined with CD19 targeting was recently reported [43]. A trial targeting CD79b with CAR T-cells is currently being initiated (NCT04609241).

Novel approaches aimed at augmenting CAR T-cell efficacy in lymphoma are ongoing. Up-regulation of checkpoints including programed death ligand-1 (PD-L1) within the tumor microenvironment of Non-Hodgkin lymphoma (NHL) patients after axicabtagene ciloleucel treatment has provided the rationale for initiating a clinical trial investigating the efficacy of CD19 CAR T-cells in combination with the anti-PD-L1 antibody Atezolizymab (Zuma-6, NCT02926833) [63]. Currently, 28 patients with diffuse large B-cell lymphoma have been treated with this combination therapy with a reported ORR of 75% and with a manageable safety profile. Other strategies to augment CAR T-cell efficacy in lymphoma include co-expression of additional co-stimulatory ligands, such as 4-1BBL [64] and CD40L [65]. Pre-clinical studies showed increased susceptibility of tumor cells to undergo apoptosis when CD40L was co-expressed on the surface of CAR T-cells. In addition, CD40L expression also increased in vivo cytotoxicity and enhanced anti-tumor immunity through up-regulation of CD80 (B7-1), CD86 (B7-2), and Fas receptor on tumor cells. Co-expressing 4-1BBL in CAR T-cells led to enhanced T-cell proliferation, IL-2 production and in vivo tumor clearance. Based on these pre-clinical findings, a phase 1 trial is currently being conducted, investigating the safety and efficacy of CD19-directed CAR T-cells co-expressing 4-1BBL (NCT03085173). The study enrolled patients diagnosed with NHL, CLL and ALL and reported an initial CR rate of 59% [66].

### T-cell leukemia and lymphoma

There are several obstacles that need to be overcome in order to successfully translate CAR T-cell therapy toward T-cell tumors. Several issues need to be overcome to ensure success of CAR T-cell therapy against T-cell malignancies including identification of proper antigens to target, fratricide, T-cell aplasia which unlike B-cell aplasia is prohibitively toxic and difficulties with CAR T-cell manufacturing, in particular the selective engineering of healthy T-cells with the CAR gene.

Only a limited number of studies targeting T-cell antigens with CAR T-cell therapy has been published (Table 1). However, common to these are that they are targeting antigens with high expression on malignant T-cell clones and no or low level expression on their healthy counterparts in order to reduce fratricide. CD30 is a member of the tumor necrosis factor receptor (TNFR) family involved in regulating T-cell proliferation and cytokine production after TCR stimulation. CD30 expression has been found on a subset of T-cell leukemia and lymphomas and with a limited expression pattern on healthy T-cells, i.e., a minor subset of activated cells. Several clinical studies are investigating the efficacy of CAR T-cells against CD30+ tumors (NCT02917083; NCT04653649; NCT03049449, NCT022595). Overall, targeting CD30 in these studies has proven to be safe [45, 60]. However, one study did report limited expansion and persistence of the infused product [60]. This observation may account for the reduced risk of developing CRS or neurotoxicity after anti-CD30 CAR T-cell therapy which is a common side effect of targeting B-cell malignancies. Besides CD30, CD37 have also been explored as target in T-cell malignancies. CD37 is a member of the tetraspanin superfamily with limited expression to the lymphoid compartment. In particular B-cells, both malignant and healthy cells have high expression of CD37 but can also be found in some T-cell lymphomas. Because CD37 is not found on healthy T-cells, anti-CD37 CAR T-cells where able to discriminate between normal and malignant T-cells and were manufactured without signs of fratricide [41]. A clinical trial evaluating CAR T-cell efficacy against CD37+ *B* and T-cell malignancies is underway (NCT04136275).

A recent study utilized CAR T-cells directed against the *β*-chain of the TCR [44]. The *β*-chain constant region can either be encoded by the T-cell receptor beta constant-1 (TRBC1) or the T-cell receptor beta constant-2 (TRBC2) gene. This means that a population of healthy T-cells will consist of a mixtures of T-cells expressing either TRBC1 or TRBC2, whereas the entire population of a T-cell cancer will exclusively express either TRBC1 or TRBC2. This knowledge can be leveraged to target the malignant T-cell clone while sparing the fraction of healthy T-cells expressing the other *β*-chain constant region. In this study CAR T-cells targeting TRBC1 could discriminate between TRBC1 and TRBC2-positive cells both in vitro and in vivo. Thus, CAR T-cells directed against either one of the *β*-chain constant regions offers a unique approach of targeting T-cell malignancies without causing complete T-cell aplasia (NCT03590574).

## Disease relapse after CAR T-cell therapy

Relapse with CD19+ tumor cells is often seen within the first months after CAR T-cell infusion and is often related to intrinsic failure of the CAR T-cells. Factors including construct design, manufacturing process, and initial cell quality and phenotype have all been suggested as crucial for sustaining CAR T-cell persistence. In a comprehensive study carried out by Fraietta and colleagues, the transcriptomic profile of CAR T-cells from complete-responding versus non-responding CLL patients was compared. T-cell products from the complete responders were enriched with T-cells with a memory-related gene signature [67]. Specifically, CAR T-cells with a central memory (*T*_CM_) or a stem cell memory (*T*_SCM_) phenotype were associated with an increased proliferative potential and persistence in patients. In contrast, relapse with CD19− tumors occurs later as result of the selective pressure by CAR T-cell therapy leading to disease relapse or selection of pre-existing malignant cell clones lacking the targeted antigen. Both mechanisms are a major obstacle for the curative potential of CAR T-cells. Antigen loss has been observed across multiple trials varying in study design, construct design, and targeted antigen. Up to 30% of patients treated with CD19 CAR T-cell therapy relapse with CD19-negative disease and several mechanisms for relapse have been proposed. These include alternative splicing events, creating truncated CD19 variants lagging the epitope recognized by CD19 CAR T-cells, outgrowth of a pre-existing CD19-negative clone, or lineage switching to myeloid leukemia. However, complete antigen loss may not be required for resistance to CAR T-cell therapy. Loss or down-regulation of target is not unique to anti-CD19 CAR T-cells, but has also been reported for other targets including CD22 and BCMA. A reduction in CD22 surface expression was sufficient to drive tumor evasion from anti-CD22 CAR T-cells despite ongoing dim CD22 expression [8].

As alternative to multi-targeting CARs, utilizing NK-cells redirected by the CAR has been proposed as alternative strategy [68, 69]. MHC class I downregulation is a common mechanism exploited by cancer cells to escape recognition by the immune system. In case of CAR-target downregulation, CAR NK-cells can facilitate tumor killing of MHC class I negative cells potentially reducing the risk of antigen-escape.

Although less understood, intrinsic CAR failure may also be related to pre-existing or treatment induced humoral and cellular immunity against the murine-derived scFv domain of the CAR [70–72]. Presence of humoral immunity against the CAR does not seem to correlate with notable effect on CAR T-cell efficacy but have been attributed to development of anaphylaxis in one patient receiving several doses of CAR T-cells [24, 70]. On the contrary, cellular immunogenicity has been suggested to hamper CAR T-cell persistence in some cases but not all [71, 73]. These observations highlight the fields’ current lack of knowledge concerning the relation between CAR design and potency to induce immunogenicity.

## What is the ideal CAR T-cell design to combat hematological cancer?

Although we have yet to fully understand all aspects of CAR T-cell functionality, we have established some features in CAR T-cell design which are important for efficacy against hematological cancers. One important factor is deciding which antigen the therapy should be directed against. The antigen should be both highly and homogeneously expressed on all tumor cells. Moreover, the antigen should be critical for survival of malignant cells in order to reduce the risk of relapse with antigen-negative tumors. The majority of CAR T-cell products currently tested (and approved) are directed against a single antigen. However, several multi-targeted CAR T-cell strategies are in the pipeline, all with the aim of circumventing disease relapse by antigen-negative tumors. The use of such multi-targeted products are envisioned to outcompete the current single targeting products in the future.

Also importantly is the choice of co-stimulatory domain in the CAR itself. Of now the majority of CARs in development employ either a CD28 or 4-1BB co-stimulatory domain to achieve full activation. The expansion kinetics of CAR T-cells harboring a CD28 co-stimulatory domain is very rapid and robust with in vivo persistence of 3 months. On the contrary, 4-1BB based CARs have slower expansion kinetics but can persist in patients for more than 5 years. These differences should be utilized when designing an optimal CAR product for various hematological cancers. As example, low tumor burden cancers like lymphoma may need to be treated with a product that can rapidly expand without significant risk of developing signs of severe CRS. On the other hand, for high tumor burden cancers, such as ALL, it might be beneficial to use CARs harboring the 4-1BB co-stimulatory domain to avoid rapid proliferation of the T-cells to reduce the risk of severe CRS and ensure long-persisting T-cells in order to eradicate all tumor cells.

The initial phenotype of CAR T-cells has also been found to be important for the efficacy of CAR T-cell therapy. In general, products enriched with T-cells having a memory phenotype have been reported to correlate with a positive outcome. The importance of cell quality over cell quantity was highlighted in a recent report. Here authors described a patient experiencing a complete response based on clonal expansion of a single CD19 CAR T-cell. Integration of the CAR gene into the T-cell genome resulted in disruption of the TET2 gene, which in turn favored a *T*_CM_ phenotype and increased the levels of perforin and granzyme in the CAR T-cell [74].

Because CAR T-cells proliferate within the patient, a linear relationship between CAR T-cell dosing and efficacy cannot easily be determined but will highly be influenced by the factors discussed above. Recently, several research groups have developed mathematical models to predict the minimum effective CAR T-cell dose needed to induce complete response [75, 76]. It will be interesting to see whether these predictions based on mathematical models will indeed be supported by clinical trials.

Currently, CAR T-cell manufacturing is a complicated, costly and time-consuming process. Much effort has been focused on key areas of the production process to ensure reliable and scalable manufacturing. However, with the rate of product approvals, the current autologous CAR T-cell manufacturing process may fail to support the increasing demand. Shifting from an autologous to allogeneic setting could ensure sustainability [77, 78]. However, even in the case where human leukocyte antigen (HLA) is matched between recipients and donors there is a still a substantial risk of graft-versus-host-disease (GvHD). To circumvent such problems manufacturing of allogeneic products has been focused on eliminating the endogenous TCR through gene-editing or utilizing cell subsets, such as NK-cells lacking natural TCR as carrier of the CAR [79]. Recently, novel approaches based on in vivo gene transfer mediated by nanocarriers or lentiviral particles have also been proposed as an attractive low-cost “off-the-shelf” strategy [80–82].

## Conclusion

In recent years, CAR T-cell therapy has proven to be an important therapy bringing new options to otherwise incurable cancer patients. Despite this, a larger fraction of patients are not cured and relapse, often with antigen-negative tumors. Discovering new antigens with a more favorable expression profile is important, however, will likely not be substantial in preventing antigen-escape. Newer approaches developing novel CAR designs targeting multiple antigens are making their way into clinical testing and have the potential for pushing the field forward. Due to the currently high costs of production, it is unlikely that CAR T-cell therapy will take the front seat as primary treatment modality over conventional therapies like surgery, chemotherapy and radiotherapy. However, new engineering approaches based on allogeneic cells products or in vivo gene delivery are on the horizon potentially providing an “of-the-shelf” solution for sustainable CAR T-cell manufacturing which finally may deliver a continuous and cheaper supply for the increasing demand.
